# Artificial Intelligence-Enabled Electrocardiography Model for Left Ventricular Systolic and Diastolic Dysfunction Predict Incident Atrial Fibrillation in Patients with Sinus Rhythm: A Time-Dependent Analysis

**DOI:** 10.3390/diagnostics16182887

**Published:** 2026-09-08

**Authors:** Kyung Su Kim, Jeong Min Son, Hak Seung Lee, Soobeen Seol, Sora Kang, Joon-myoung Kwon, Min Sung Lee, Kyung-Hee Kim

**Affiliations:** 1 Medical AI Co., Ltd., Seoul 06180, Republic of Korea; kanesu@medicalai.com (K.S.K.); jmson@medicalai.com (J.M.S.); cardiolee@gmail.com (H.S.L.); soobeen@medicalai.com (S.S.); kangsora39@medicalai.com (S.K.); jm.kwon@medicalai.com (J.-m.K.); 2 Digital Healthcare Institute, Sejong Medical Research Institute, Bucheon 14754, Republic of Korea; 3 Division of Cardiology, Department of Internal Medicine, Incheon Sejong Hospital, Cardiovascular Center, Incheon 21080, Republic of Korea; learnbyliving9@gmail.com

**Keywords:** artificial intelligence, electrocardiography, atrial fibrillation, diastolic dysfunction, heart failure

## Abstract

**Background/Objectives:** Artificial intelligence-enabled electrocardiography (AI-ECG) can infer left ventricular systolic dysfunction (LVSD) and diastolic dysfunction (LVDD) from a standard 12-lead tracing. Whether these structural AI-ECG scores predict incident atrial fibrillation (AF) in patients in sinus rhythm, and how their predictive value changes over time, is unclear. **Methods:** In a retrospective single-center cohort of patients with a sinus-rhythm index ECG, AI-ECG LVSD and LVDD scores were derived and dichotomized at prespecified cutoffs (LVSD ≥ 9.7; LVDD ≥ 20.8). The outcome was incident AF, assessed within 30, 90, 180, 270, and 365 days. Discrimination was quantified by the AUROC. Because the proportional hazards assumption was violated, the time course of risk for the joint LVSD × LVDD classification was characterized with window-specific Cox, Aalen additive hazards, and restricted mean survival time analyses. **Results:** Among 19,593 index ECGs from 18,984 patients, 1190 (6.07%) were followed by incident AF within one year. One-year incidence rose from 4.1% (both negative) to 9.8% (LVSD-positive only), 20.1% (LVDD-positive only), and 19.9% (both positive). Discrimination was highest for early events and attenuated over time, more steeply for LVSD (AUROC 0.803 at 30 days to 0.713 at 365 days) than LVDD (0.817 to 0.764). Combining AI-ECG LVSD and LVDD models, the excess risk conferred by dual positivity was concentrated in the first weeks after the index ECG and, consistently across cumulative Cox, Aalen additive hazards, and restricted mean survival time analyses, converged with that of isolated LVDD positivity by one year. **Conclusions:** Structural AI-ECG LVSD and LVDD scores from a single sinus-rhythm ECG predict incident AF in a time-dependent manner, with the strongest performance shortly after acquisition and more durable discrimination for LVDD. A single AI-ECG may help target short-term AF surveillance, particularly in patients with combined systolic–diastolic dysfunction.

## 1. Introduction

Atrial fibrillation (AF) is the most common cardiac arrhythmia and a major cause of stroke, heart failure, and death [1,2,3,4]. A substantial fraction of AF is diagnosed only after a thromboembolic event, so tools that identify high-risk patients while in sinus rhythm could enable earlier surveillance and preventive care [5,6]. Established clinical scores capture demographic and comorbidity burden but do not directly interrogate the atrial and ventricular substrate that precedes AF [7,8]. Recent studies have shown that deep learning applied to standard 12-lead electrocardiography (ECG) recorded during sinus rhythm can identify subtle electrophysiological signatures to predict incident AF, and that artificial intelligence enabled ECG (AI ECG)-guided screening significantly increases the clinical detection rate of AF [9,10,11].

Structural heart disease—particularly left ventricular systolic dysfunction (LVSD) and diastolic dysfunction (LVDD)—is mechanistically linked to atrial remodeling and subsequent electroanatomical substrates, thereby predisposing patients to incident AF [12,13,14,15,16]. Early AI ECG models focused on the ability to detect such structural alterations from standard 12-lead ECGs, leading to their integration into real-world clinical practice [17,18,19,20]. However, the predictive value of AI ECG models targeting structural heart disease for incident AF has not been fully established. We hypothesized that AI ECG models for LVSD and LVDD could serve as predictors for incident AF. Accordingly, we evaluated whether positive findings from these structural AI ECG models were associated with subsequent AF.

## 2. Materials and Methods

### 2.1. Study Design and Population

This study was a retrospective analysis performed in a single institution. All 12-lead ECGs from patients aged 18 years or older between 2021 and 2024 were screened for enrollment. To be eligible, a patient had to be in sinus rhythm with no prior clinical diagnosis of AF (ICD-10 I48) on or before the index ECG; patients with a history of AF were excluded. Because outcome ascertainment required linking an index ECG to subsequent follow-up, each eligible patient had to have at least two 12-lead ECGs during the study period—one or more sinus-rhythm ECGs serving as the index ECG(s) and at least one later ECG to determine the outcome. Patients were excluded for any of the following: fewer than two ECGs during the study period (no follow-up ECG available to ascertain the outcome); a pacing rhythm on the ECG; cardiac surgery within 2 weeks of the ECG; AF on all available ECGs; a discrepancy between the clinical diagnosis and the ECG rhythm (an AF diagnosis without AF on any ECG, or AF on an ECG without a corresponding AF diagnosis); or any rhythm other than sinus rhythm or AF.

Each patient contributed one or more sinus-rhythm 12-lead ECGs (the index ECG) linked to subsequent follow-up. Patients with a first AF diagnosis within 365 days formed the incident AF group, and those without AF and with at least one follow-up sinus-rhythm ECG within 365 days formed the control group. For patients in the AF group, every qualifying sinus-rhythm ECG recorded before the first AF diagnosis was used as an index ECG—so a patient with several eligible ECGs contributed multiple index ECGs, each retaining its own interval to the subsequent AF diagnosis (0–364 days). For controls, the earliest qualifying sinus-rhythm ECG was used as a single index ECG, with follow-up censored at 365 days. Throughout enrollment and analysis, atrial flutter was regarded as AF.

The study was approved by the institutional review board (IRB No 2024-09-001-006), and written informed consent was waived due to the retrospective nature of the study. All activities involving human participants were conducted in accordance with the ethical standards of the institutional review board and Declaration of Helsinki and its later amendments.

### 2.2. AI–ECG Models

Two previously developed deep learning models were applied to the index 12-lead ECG without retraining or recalibration. The original AI–ECG LVSD model was a convolutional neural network-based model for detecting LVSD defined as left ventricular ejection fraction of 40% or less on echocardiography (AiTiA LVSD version 1.00.00, Medical AI Co., Ltd., Seoul, Republic of Korea) [18]. We have utilized an updated version (AiTiA LVSD version 2.00.00, Medical AI Co., Ltd., Seoul, Republic of Korea), which is based on a foundation model and employs a hybrid architecture as follows: convolutional encoders extract local waveform morphologies, while transformer–based encoder captures long–range temporal dependencies and global rhythm characteristics through self-attention mechanisms [21,22]. The algorithm generates a probability score for LVSD between 0 and 100 using standard 12-lead ECG signals at 500 Hz. A pre-established high-risk threshold is 9.7, which was optimized for screening purposes. This algorithm was extensively validated in various clinical environments [23,24,25,26]. AI–ECG LVDD model (AiTiA LVDD version 1.00.00, Medical AI Co., Ltd., Seoul, Republic of Korea) is built upon a foundation model and fine-tuned to detect an increased E/e’ on echocardiography [20]. AI–ECG LVDD model also generates a probability score for LVDD between 0 and 100 with a predetermined high-risk threshold of 20.8.

The high-risk thresholds used in this study (LVSD ≥ 9.7; LVDD ≥ 20.8) were pre-specified and fixed before the present analysis and were not derived from the current cohort. The LVSD threshold of 9.7 is the established screening cutoff reported in prior development and validation studies [22,23] and has been in use since the model was approved as software as a medical device (SaMD) by the Korean Ministry of Food and Drug Safety (MFDS) in March 2023. For the LVDD model, an earlier report derived a Youden-index cutoff of 13.3 [20]; however, the deployed model was developed in May 2025 and, since receiving MFDS SaMD approval in November 2025, has used a fixed high-risk threshold of 20.8, which is the value applied in the present study. In all cases, the models and their thresholds were applied to the index ECGs without retraining, recalibration, or threshold re-optimization.

### 2.3. Outcome

**Figure 1:**
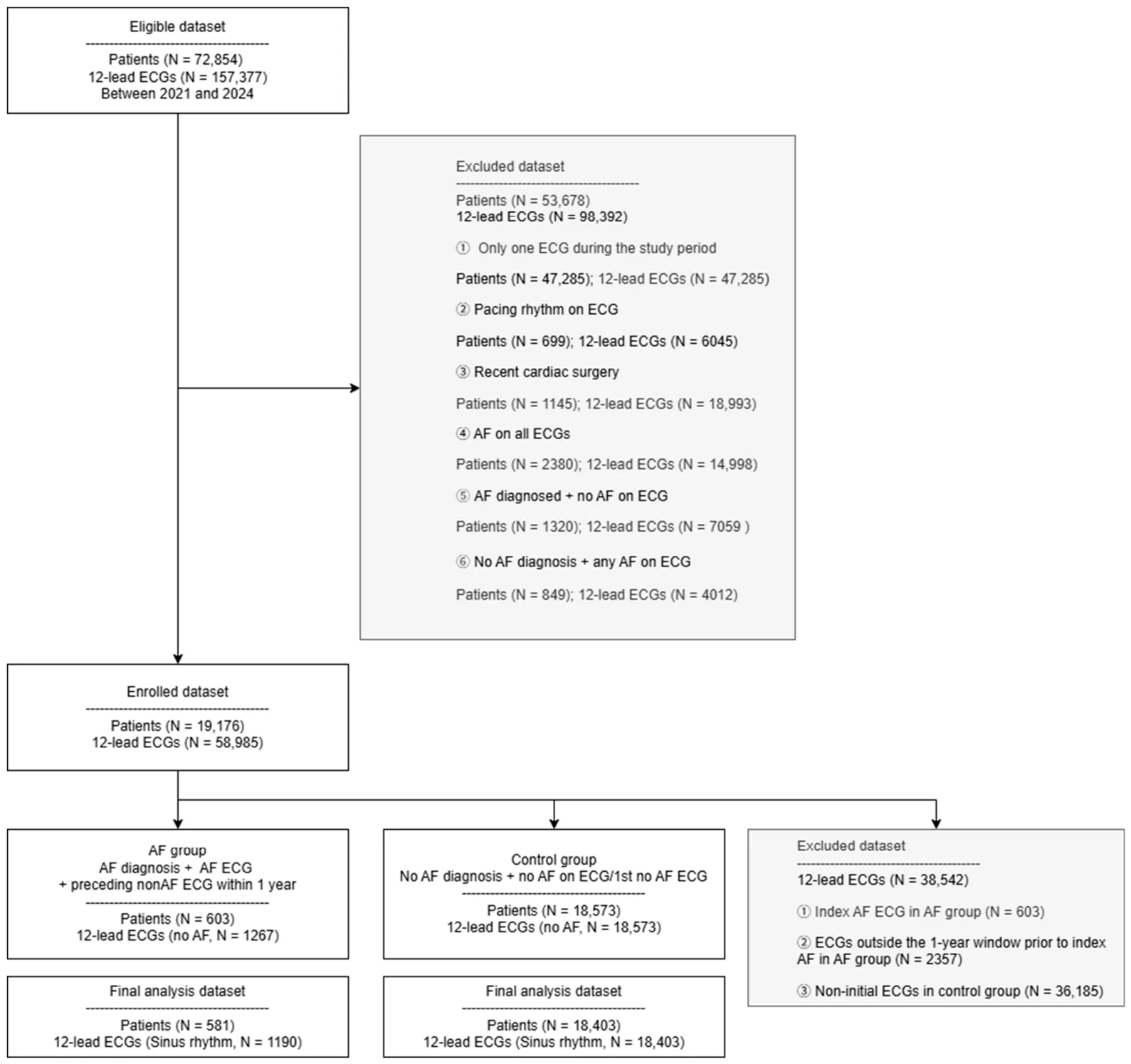
Study enrollment flowchart. Of 72,854 patients (157,377 12-lead ECGs) recorded between 2021 and 2024, 53,678 patients (98,392 ECGs) were excluded for the following reasons: only one ECG during the study period; a pacing rhythm on ECG; recent cardiac surgery; atrial fibrillation (AF) on all ECGs; an AF diagnosis without AF on ECG; or no AF diagnosis but AF on ECG. The enrolled dataset comprised 19,176 patients (58,985 ECGs), classified into an AF group (603 patients with an AF diagnosis, an AF-showing ECG, and at least one preceding non-AF ECG; 603 AF and 1267 non-AF ECGs) and a control group (18,573 patients without AF; 18,573 ECGs). After restriction to sinus-rhythm index ECGs, the final analysis dataset comprised 581 patients with 1190 sinus-rhythm ECGs in the AF group and 18,403 patients with 18,403 sinus-rhythm ECGs in the control group. AF, atrial fibrillation; ECG, electrocardiogram.

Incident AF was defined as a new diagnosis of AF—in a patient with no prior AF—documented on a follow-up 12-lead ECG within 365 days of the index ECG and corroborated by a corresponding AF diagnosis code (ICD-10 I48) in the electronic health record. The cardiac rhythm was determined from the standard 12-lead ECG using the device’s automated rhythm interpretation, and atrial flutter was regarded as AF. An AF event required documentation of AF on a 12-lead ECG; ambulatory or continuous rhythm monitoring (e.g., Holter or patch ECG) was not used for outcome ascertainment. To ensure concordance between the recorded rhythm and the clinical diagnosis, patients with a discrepancy between the two were excluded at enrollment (Section 2.1; Figure 1). For cases, the time from the index ECG to the AF diagnosis was recorded (0–364 days); for controls, follow-up was censored at 365 days.

The primary outcome was defined as incident AF at 365 days. The secondary outcome was defined as incident AF at various follow-up durations (30, 90, 180, and 270 days) to investigate the temporal relationship between the structural AI–ECG model and incident AF.

### 2.4. Variables

Age at the index ECG and sex were obtained. Underlying diseases including hypertension, diabetes, coronary artery disease, cerebrovascular disease, congestive heart failure, chronic kidney disease, and peripheral artery disease were identified using ICD-10 diagnosis codes. CHA2DS2-VASc score was calculated using age, sex, and underlying diseases. Standard ECG features including heart rate, PR interval, QRS duration, corrected QT interval, and T-wave axis were recorded. Continuous variables without a recorded value were treated as missing and handled by available-case analysis, whereas the absence of a qualifying ICD-10 diagnosis code was interpreted as absence of the corresponding condition. Missing data were minimal and confined to the following three ECG-derived measurements: T-wave axis (145 ECGs, 0.74%), PR interval (25, 0.13%), and QTc interval (1, 0.01%). Age, sex, comorbidities, and the derived CHA2DS2-VASc score—ascertained from EHR diagnosis codes—and the AI-ECG LVSD and LVDD scores had no missing values. Variables with missing values were handled by available-case analysis.

### 2.5. Statistical Analysis

Continuous variables were described as medians with interquartile ranges (IQRs) and categorical variables were expressed as numbers with percentages. Continuous variables between two groups were compared using the Wilcoxon rank-sum test and categorical variables between two groups were compared using Chi-square analysis.

Each AI–ECG model score was analyzed in the following two ways: as a continuous variable and as a binary variable using the prespecified cutoffs (LVSD ≥ 9.7; LVDD ≥ 20.8). AF prevalence across various prediction windows was compared by AI–ECG model score bins and displayed as heatmap plot with marginal bar graph. The discriminative ability of AI–ECG LVSD and LVDD scores was tested using area under the receiver operating characteristics curve (AUROC) with 95% confidence intervals (CIs) and paired comparisons by the DeLong method. AI–ECG model positivity was tested using Cox proportional hazard regression estimated hazard ratios (HRs) and cumulative incidence was displayed using Kaplan–Meier analysis. Single model positivity was tested separately and joint four-group classification (both negative; LVSD positive only; LVDD positive only; and both positive) was analyzed. Because the LVSD and LVDD thresholds were developed to detect structural dysfunction rather than to predict AF, the cutoff-based analyses (score positivity and the four-group classification) should be interpreted as clinically convenient dichotomizations rather than AF-optimized decision thresholds. The primary discrimination (AUROC) and dose–response analyses were based on the continuous scores and are therefore independent of these cutoffs.

To evaluate the incremental value of the AI-ECG scores beyond established clinical risk factors, we calculated the CHA2DS2-VASc score—which incorporates age, sex, hypertension, diabetes mellitus, heart failure, prior stroke/thromboembolism, and vascular disease—for each patient and used it as the clinical benchmark. For AF within 30 and 365 days, we compared logistic models as follows: CHA2DS2-VASc alone versus CHA2DS2-VASc plus the AI-ECG LVSD score, the LVDD score, or both. Incremental discrimination was assessed by the change in AUROC (ΔAUROC) with the DeLong test, and incremental reclassification by the category-free net reclassification improvement (NRI) and integrated discrimination improvement (IDI), with 95% confidence intervals from 1000 bootstrap resamples.

The proportional hazard assumption for the joint four-group classification was assessed using scaled Schoenfeld residuals (Kaplan–Meier and rank-transformed time). The assumption was significantly violated for both positive (χ^2^(1) = 30.6 and 31.0, both transformations *p* < 0.005) and LVSD positive only (χ^2^(1) = 9.35 and 9.38, both transformations *p* < 0.005) and was borderline for LVDD positive only (χ^2^(1) = 3.95 and 3.98, *p* = 0.05), indicating that the hazard ratio for AI–ECG risk group was not constant over the follow-up period. Because of this violation, the time course of this hazard difference was analyzed using three complementary cumulative approaches. First, cumulative hazard ratios relative to the both-negative reference were estimated from Cox models with follow-up censored at 30, 90, 180, 270, and 365 days, each representing the average hazard ratio for incident AF over the corresponding window. Second, Aalen’s non-parametric additive hazard model estimated the cumulative excess-hazard function for each group relative to the reference without assuming any specific functional form for its time-dependence. Third, the restricted mean survival time (RMST), defined as the mean AF-free time up to a prespecified truncation point τ, was estimated for each group at τ = 30, 90, 180, 270, and 365 days, with between-group differences and 95% confidence intervals derived from 500 bootstrap resamples. As a sensitivity analysis, a time-varying Cox model incorporating an interaction between the four-group classification and the logarithm of follow-up time was fitted to estimate the instantaneous hazard ratio across follow-up.

The association between each continuous AI–ECG score and the hazard of incident AF was further modeled using restricted cubic splines (4 degrees of freedom) within an unadjusted Cox proportional-hazard framework, fitted separately for 30-day and 1-year incident AF. The hazard ratio at each value of the score was expressed relative to the cohort median score, with 95% confidence intervals derived from the model covariance matrix; a small ridge penalty (λ = 0.01) was applied to stabilize estimation given collinearity among the spline basis terms. The frequency distribution of each score was displayed as an overlaid histogram.

To assess whether the association between the AI-ECG scores and incident AF was driven by events that were already imminent or subclinically present at the index ECG, we performed a prespecified sensitivity analysis using blanking periods. AF events with time from index ECG < 7 days or <14 days were excluded, and the analyses were repeated in the remaining sample for both the 30-day and 1-year outcomes. For each blanking period and outcome, we re-computed the discrimination (AUROC) of the continuous LVSD and LVDD scores and re-estimated unadjusted Cox hazard ratios for LVSD and LVDD positivity at the prespecified cutoffs (LVSD ≥ 9.7; LVDD ≥ 20.8).

The primary analysis was performed at the ECG level as follows: in the AF group, 581 patients contributed 1190 sinus-rhythm index ECGs, each linked to the subsequent AF event, whereas controls contributed a single index ECG each. To assess the influence of within-patient clustering, we performed a sensitivity analysis restricted to one index ECG per patient (the earliest sinus-rhythm ECG), yielding 18,984 patients (581 with incident AF, 3.06%), and compared the patient-level and ECG-level AUROCs using 1000 patient-level (cluster) bootstrap resamples.

Robustness was assessed by the consistency of discrimination and effect estimates with those of the primary analysis. Subgroup analysis defined by age (<60 vs. ≥60 years), sex, and underlying disease was performed. Analyses were performed in Python 3 (pandas, numpy, scipy, statsmodels, patsy, scikit-learn, and lifelines) and all tests were two-sided with *p* < 0.05 considered significant.

## 3. Results

### 3.1. Participant Enrollment

Between 2021 and 2024, 72,854 patients with 157,377 12-lead ECGs were eligible. We excluded 53,678 patients (98,392 ECGs) for having only one ECG during the study period, a pacing rhythm, recent cardiac surgery, AF on all ECGs, or a discrepancy between the AF diagnosis and the ECG rhythm, leaving an enrolled dataset of 19,176 patients (58,985 ECGs) (Figure 1). These comprised an AF group—603 patients with an AF diagnosis, an AF-showing ECG, and at least one preceding non-AF ECG—and a control group of 18,573 patients without AF. After restricting to sinus-rhythm index ECGs, the final analytic sample comprised 19,593 index ECGs as follows: 1190 from 581 patients who developed incident AF and 18,403 from 18,403 control patients.

### 3.2. Participant Characteristics

Among 19,593 index ECGs in sinus rhythm, 1190 (6.07%) were followed by incident AF within one year. Compared with controls, patients who developed AF were older, more often male (both *p* < 0.001). They also had a markedly higher burden of comorbidity, including hypertension (65.8% vs. 39.6%), congestive heart failure (69.7% vs. 15.5%), diabetes (24.9% vs. 13.3%), coronary artery disease (34.3% vs. 26.6%), chronic kidney disease (16.1% vs. 4.4%), and cerebrovascular disease (13.9% vs. 6.0%), and a higher CHA_2_DS_2_-VASc score (median 3 vs. 1; all *p* < 0.001). On the index ECG, they had slower heart rate, longer PR intervals, wider QRS duration, and longer QTc (all *p* < 0.001). All AI-ECG scores were significantly higher in patients who developed AF (Table 1). The same pattern was more pronounced for AF within 30 days, with a higher prevalence of AI-ECG positivity (both positive, 23.0%) and older age but no sex difference (Supplementary Table S1).

### 3.3. AF Incidence Across AI-ECG LVSD and LVDD Score Bins

**Figure 2:**
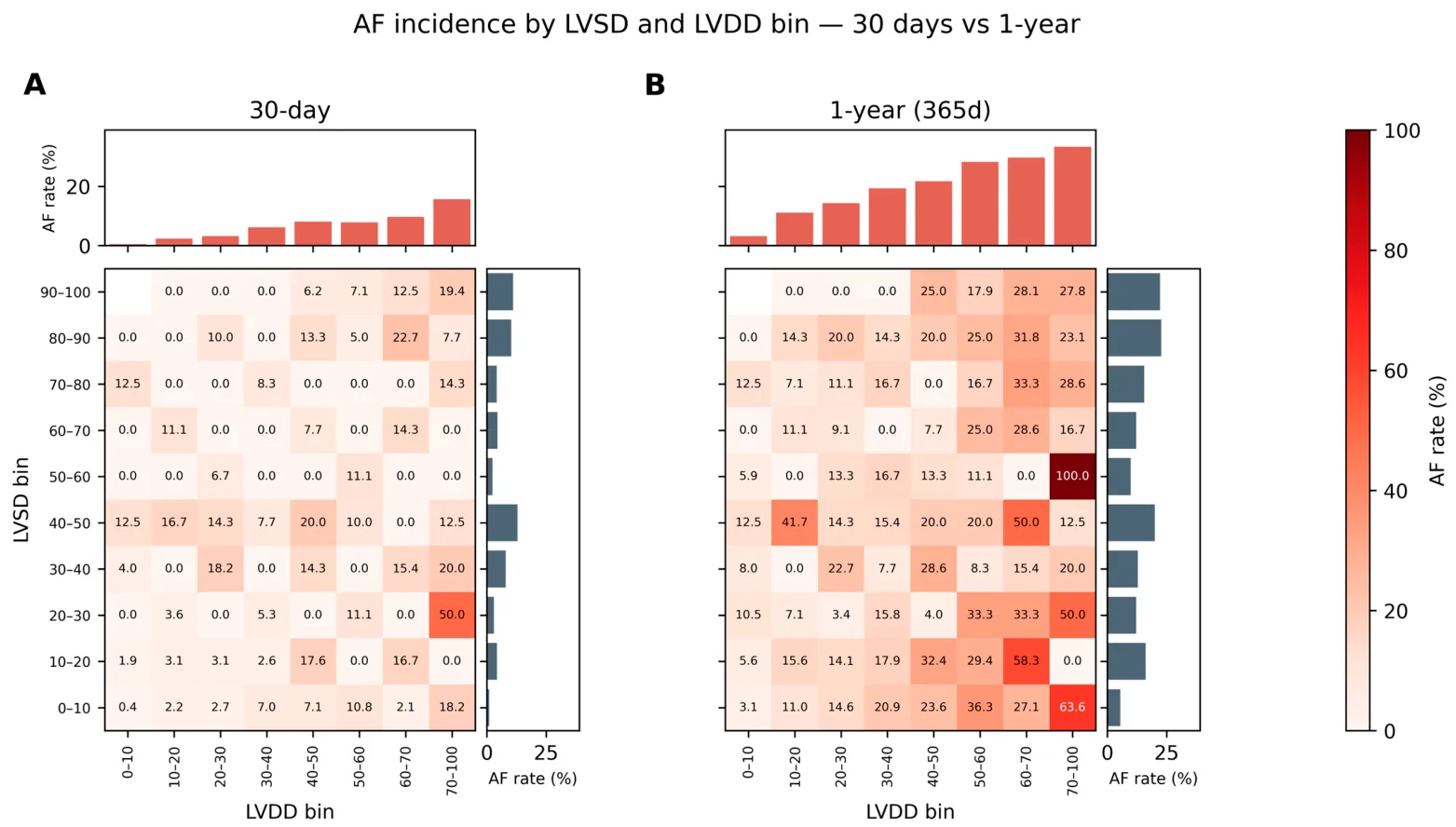
AF incidence across joint bins of the AI-ECG LVSD and LVDD scores. Heatmaps show the observed incidence of atrial fibrillation (AF, %) within each combination of LVSD (rows) and LVDD (columns) score bins, for AF occurring within 30 days (**A**) and within 1 year (365 days) (**B**) of the index electrocardiogram. LVSD was binned in 10-point intervals; LVDD was binned in 10-point intervals up to 70 with a combined 70–100 bin owing to sparse data at the highest scores. Cell shading is proportional to AF incidence (color scale, (**right**)). Marginal bar charts show AF incidence by LVDD bin (**top**) and by LVSD bin (**right**). AF incidence increased predominantly along the LVDD axis, with a weaker gradient along the LVSD axis. Cells in the highest score bins contain small numbers of patients, and the corresponding incidence estimates (including cells of 0% or 100%) are therefore unstable; blank cells contain no patients. AF, atrial fibrillation; AI-ECG, artificial intelligence-enabled electrocardiography; LVDD, left ventricular diastolic dysfunction; LVSD, left ventricular systolic dysfunction.

To visualize the crude relationship between the two AI-ECG scores and AF risk, we cross-tabulated AF incidence across score bins of the LVSD and LVDD scores (Figure 2). LVSD was divided into 10-point bins; LVDD was divided into 10-point bins up to 70 and a combined 70–100 bin, because few patients had LVDD scores above 80. AF incidence increased steeply and monotonically along the LVDD axis as follows: across LVDD bins from 0 to 10 to 70–100, 30-day incidence rose from 0.5% to 15.7% (Figure 2A) and one-year incidence from 3.2% to 33.3% (Figure 2B). Along the LVSD axis the gradient was weaker and less consistent, with one-year incidence rising only modestly and non-monotonically across bins. One-year incidence exceeded 30-day incidence throughout, and the highest-risk region of the map lay at high LVDD values largely irrespective of the LVSD value.

### 3.4. Discrimination of AI–ECG Scores Across Follow-Up Durations

**Figure 3:**
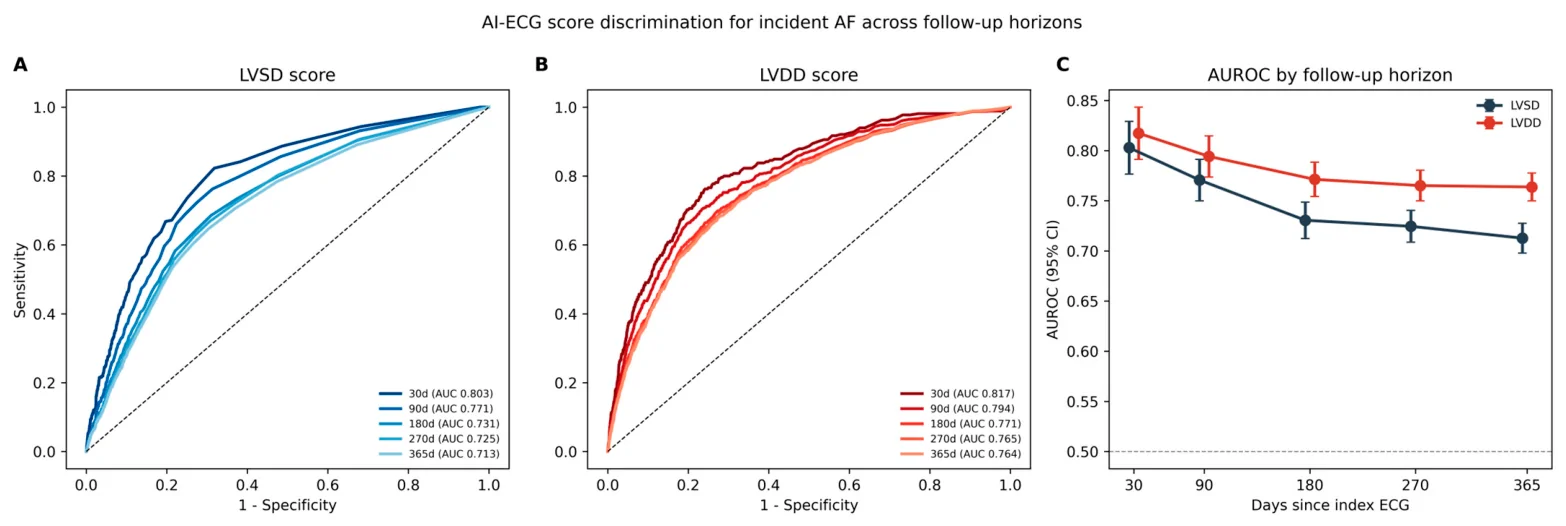
Discrimination of AI-ECG LVSD and LVDD scores for incident atrial fibrillation across follow-up durations. Receiver operating characteristic (ROC) curves for the AI-ECG LVSD score (**A**) and LVDD score (**B**) predicting incident atrial fibrillation (AF) occurring within 30, 90, 180, 270, and 365 days of the index electrocardiogram. Areas under the curve (AUROC) are shown in each panel. (**C**) AUROC as a function of follow-up horizon for the two scores; symbols denote point estimates and error bars the 95% confidence intervals. For both scores, discrimination was highest for short-term events and attenuated as the horizon lengthened. AF, atrial fibrillation; AI-ECG, artificial intelligence-enabled electrocardiography; AUROC, area under the receiver operating characteristic curve; CI, confidence interval; LVDD, left ventricular diastolic dysfunction; LVSD, left ventricular systolic dysfunction; ROC, receiver operating characteristic.

Both AI-ECG scores discriminated incident AF at every follow-up horizon, with highest AUROC at 30-day and decreasing AUROC according to follow-up periods. For the LVSD score, AUROC declined from 0.803 for AF within 30 days to 0.713 at 365-day. The LVDD score showed the same pattern but with less attenuation, from 0.817 at 30 days to 0.764 at 365 days, respectively (Figure 3A,B, Table 2). Consequently, the two scores performed comparably for very short-term prediction, whereas the LVSD–LVDD gap widened over time because discrimination degraded more steeply for the LVSD score (ΔAUROC −0.090 vs. −0.053 from 30 days to 365 days) (Figure 3C, Table 2).

### 3.5. Incremental Value of AI–ECG Scores Beyond the CHA2DS2-VASc Score

**Figure 4:**
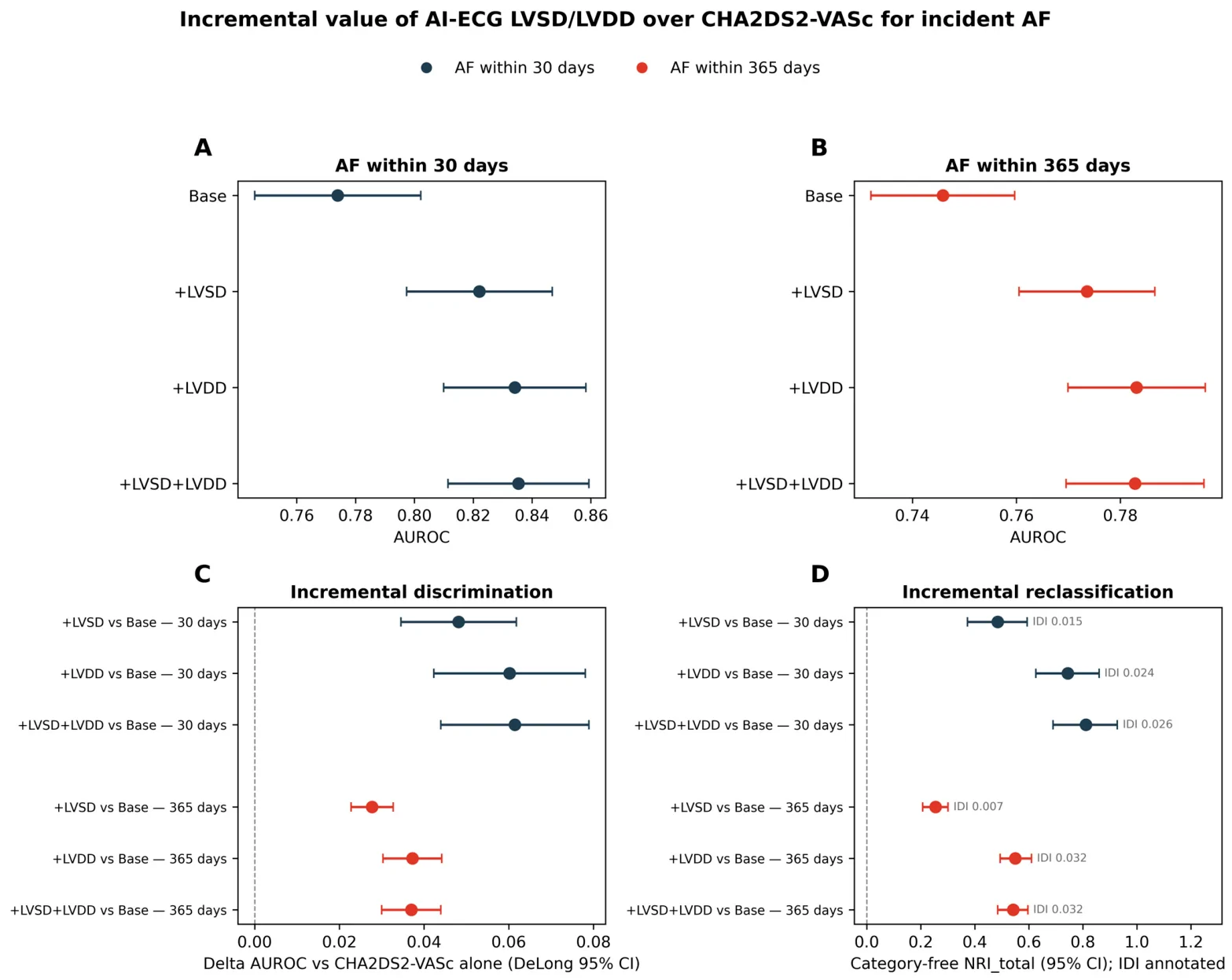
Incremental value of the AI-ECG LVSD and LVDD scores over the CHA_2_DS_2_-VASc score for incident AF. (**A**,**B**) AUROC (95% CI) of the CHA2DS2-VASc score alone (Base) and with the LVSD score, the LVDD score, or both added, for AF within 30 days (**A**) and within 1 year (**B**). (**C**) Change in AUROC (ΔAUROC) relative to CHA2DS2-VASc alone, with 95% CI estimated by the DeLong method. (**D**) Category-free net reclassification improvement (NRI, 95% CI) for each score added to CHA2DS2-VASc, with the integrated discrimination improvement (IDI) annotated. Estimates for reclassification metrics were obtained from 1000 bootstrap resamples. AF, atrial fibrillation; AI-ECG, artificial intelligence-enabled electrocardiography; AUROC, area under the receiver operating characteristic curve; IDI, integrated discrimination improvement; LVDD/LVSD, left ventricular diastolic/systolic dysfunction; NRI, net reclassification improvement.

The CHA2DS2-VASc score alone discriminated incident AF with an AUROC of 0.774 (95% CI, 0.746–0.802) at 30 days and 0.746 (0.732–0.760) at 1 year (Figure 4A,B). Adding the AI-ECG LVDD score significantly improved discrimination at both horizons—to 0.834 (0.810–0.858) at 30 days (ΔAUROC 0.060; DeLong *p* < 0.001) and 0.783 (0.770–0.796) at 1 year (ΔAUROC 0.037; *p* < 0.001) (Figure 4C)—and improved reclassification, with a category-free NRI of 0.744 (0.627–0.865) and IDI of 0.024 (0.017–0.031) at 30 days, and NRI 0.550 (0.492–0.607) and IDI 0.032 (0.027–0.037) at 1 year (Figure 4D). The LVSD score also added discrimination (ΔAUROC 0.048 at 30 days, 0.028 at 1 year; both *p* < 0.001) but with smaller reclassification gains (IDI 0.015 and 0.007). For both scores the NRI was driven predominantly by correct down-classification of non-events, with a near-null or negative event-level NRI (LVSD NRI_events −0.389 at 30 days and −0.595 at 1 year). Adding both scores together provided no meaningful improvement beyond CHA_2_DS_2_-VASc plus the LVDD score (30-day AUROC 0.835 vs. 0.834; 1-year 0.783 vs. 0.783; and IDI 0.026 vs. 0.024 and 0.032 vs. 0.032). Thus, the AI-ECG LVDD score provided incremental discrimination and reclassification for incident AF beyond the CHA_2_DS_2_-VASc score and accounted for essentially all the incremental value of the two AI-ECG scores combined (Supplementary Tables S2 and S3).

### 3.6. Cumulative Incidence and Hazard of AF by Cutoff-Defined Groups

**Figure 5:**
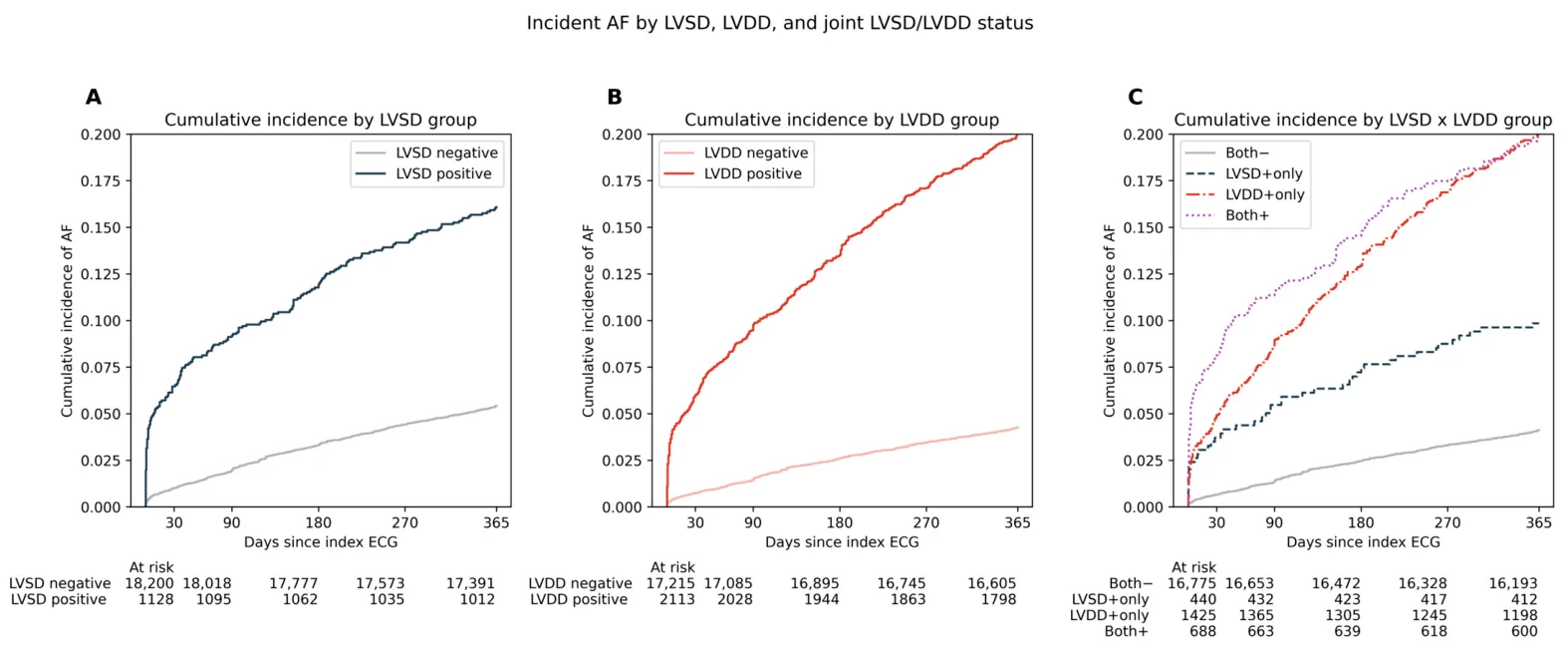
Kaplan–Meier estimated incidence of AF over one year by (**A**) AI–ECG LVSD, (**B**) AI–ECG LVDD, and (**C**) joint AI–ECG LVSD × AI–ECG LVDD group (both negative; LVSD-positive only; LVDD-positive only; and both positive). Numbers at risk for each group are shown below the *x*-axis. AF, atrial fibrillation; AI–ECG, artificial intelligence-enabled electrocardiography; LVSD, left ventricular systolic dysfunction; LVDD, left ventricular diastolic dysfunction.

Using the prespecified cutoffs (LVSD ≥ 9.7, LVDD ≥ 20.8), one-year cumulative incidence of AF was higher in score-positive patients as follows: 16.1% in LVSD-positive versus 5.4% in LVSD-negative (Figure 5A), and 20.0% in LVDD-positive versus 4.3% in LVDD-negative (Figure 5B). In the joint four-group classification, one-year cumulative incidence rose from 4.1% in the both-negative group to 9.8% in the LVSD-positive-only group, 20.1% in the LVDD-positive-only group, and 19.9% in the both-positive group (Figure 5C).

### 3.7. Time-Dependent Analysis

**Figure 6:**
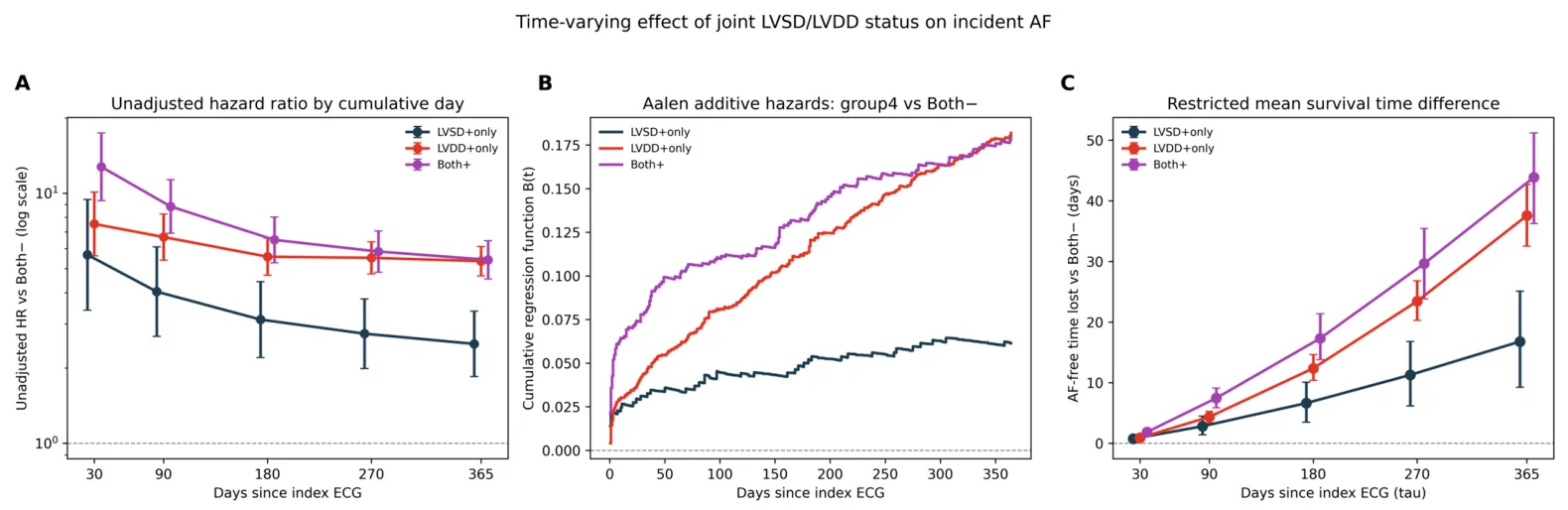
Time course of the association between joint AI-ECG LVSD × LVDD status and incident AF over one year, characterized with three complementary cumulative approaches given violation of the proportional-hazard assumption for the four-group classification. (**A**) Cumulative hazard ratios for LVSD-positive-only, LVDD-positive-only, and both-positive groups relative to the both-negative reference, each estimated from a Cox model with follow-up censored at 30, 90, 180, 270, and 365 days; error bars represent 95% confidence intervals. Each estimate is the average hazard ratio for incident AF over the corresponding window. (**B**) Cumulative excess hazard B(t) for each group relative to the both-negative reference, estimated with Aalen’s non-parametric additive hazard model without assuming a functional form for its time-dependence. (**C**) Difference in restricted mean survival time (AF-free days lost) for each group relative to the both-negative reference at truncation points τ = 30, 90, 180, 270, and 365 days; error bars represent 95% confidence intervals from 500 bootstrap resamples. All three approaches consistently show that the excess AF risk conferred by combined LVSD/LVDD positivity was concentrated in the early period after the index ECG and converged toward that of isolated LVDD positivity by one year. AF, atrial fibrillation; AI-ECG, artificial intelligence-enabled electrocardiography; LVSD, left ventricular systolic dysfunction; LVDD, left ventricular diastolic dysfunction.

Because the proportional-hazard assumption was violated for the joint four-group classification, we characterized the time course of risk using three complementary cumulative approaches (Figure 6). In the windowed cumulative Cox analysis (Figure 6A, Supplementary Table S4), the hazard ratio relative to the both-negative reference was greatest in the earliest window and declined progressively for all groups. The both-positive group had the highest early hazard (HR 12.75; 95% CI, 9.34–17.41 at 30 days) and declined to converge with the LVDD-positive-only group by one year (HR 5.42; 4.54–6.46 vs. 5.35; 4.67–6.12), whereas the LVSD-positive-only group carried the lowest hazard throughout (5.67 at 30 days to 2.50 at 365 days). Aalen’s additive hazard model (Figure 6B) corroborated this pattern as follows: the cumulative excess hazard for the both-positive group rose most steeply within the first 2–3 weeks after the index ECG, after which its slope flattened and ran nearly parallel to that of the LVDD-positive-only group, the two converging to a similar cumulative excess hazard by one year (B(365) ≈ 0.18 for both), while the LVSD-positive-only group plateaued at a substantially lower level (B(365) ≈ 0.06). Consistent with these findings, the RMST analysis (Figure 6C) showed that by one year the both-positive, LVDD-positive-only, and LVSD-positive-only groups had lost a mean of 43.9, 37.6, and 16.8 AF-free days, respectively, relative to the both-negative reference; the incremental AF-free time lost by the both-positive group relative to the LVDD-positive-only group accrued predominantly within the first months of follow-up and did not increase further thereafter. Together, these three independent approaches consistently demonstrated that the excess risk of incident AF conferred by combined LVSD/LVDD positivity was concentrated early after the index ECG and converged cumulatively toward that of isolated LVDD positivity by one year.

As a sensitivity analysis, a single time-varying Cox model with a four-group × log(time) interaction yielded instantaneous hazard ratios that reproduced the same temporal pattern (Supplementary Figure S1). The both-positive and LVDD-positive-only groups had similar hazards at 30 days (HR 3.81 and 3.67 vs. both-negative), but the both-positive estimate fell below the LVDD-positive-only estimate after approximately 2–3 months and remained lower through 365 days (HR 2.04 vs. 2.55); the LVSD-positive-only group was lowest throughout (1.93 to 1.29). Absolute magnitudes differ from the windowed cumulative estimates (Figure 6A) because the two models estimate different quantities (window-averaged versus instantaneous hazard ratios), but the temporal pattern was concordant.

### 3.8. Dose–Response Relationship of Continuous AI-ECG Scores

**Figure 7:**
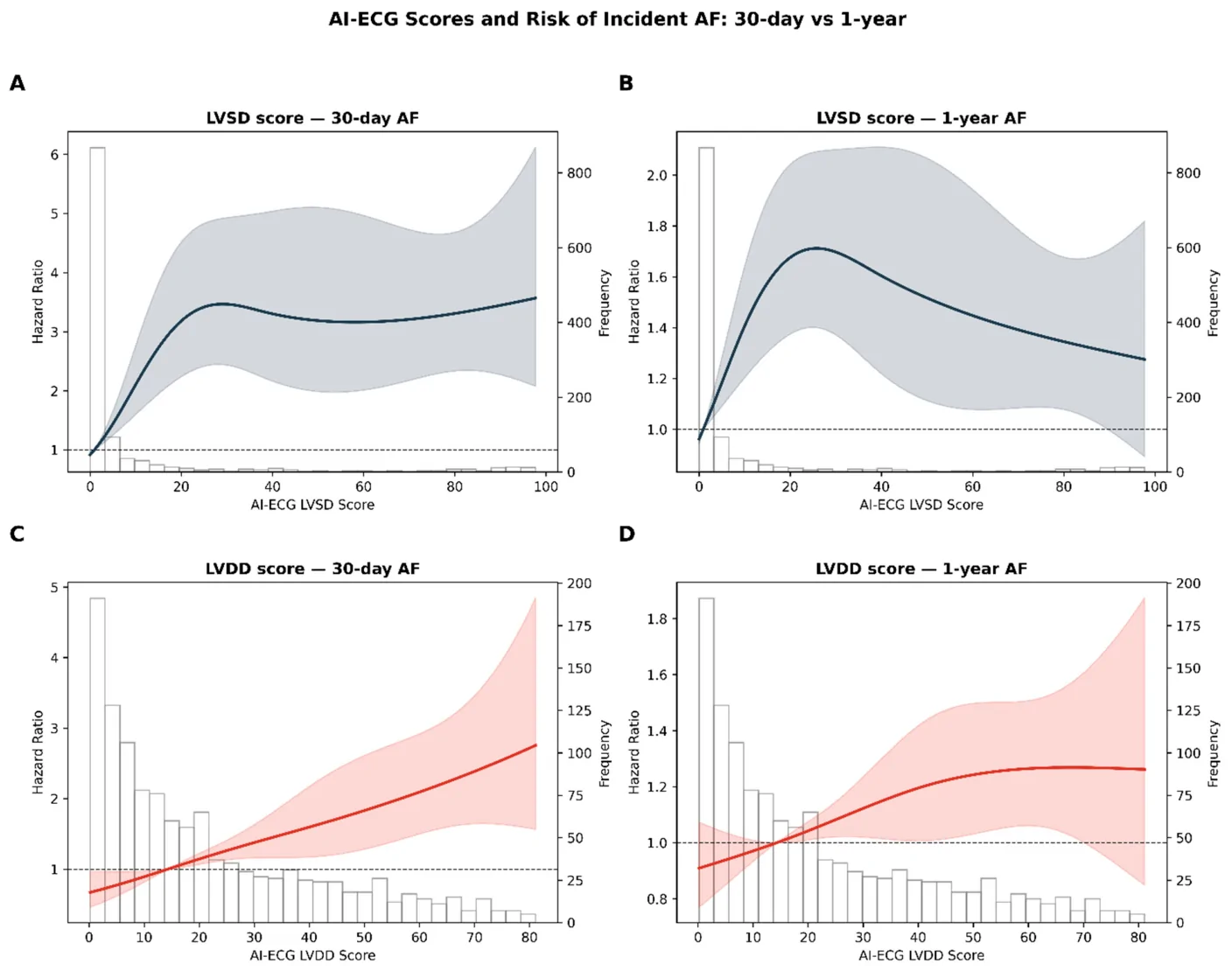
Dose–response association between continuous AI-ECG scores and incident AF at 30 days and 1 year. Unadjusted restricted cubic spline Cox models showing the hazard ratio (HR) for incident atrial fibrillation (AF) as a continuous function of AI-ECG scores relative to the cohort median: (**A**) AI-ECG LVSD score for AF occurring within 30 days; (**B**) AI-ECG LVSD score for AF occurring within 1 year (365 days); (**C**) AI-ECG LVDD score for AF occurring within 30 days; and (**D**) AI-ECG LVDD score for AF occurring within 1 year (365 days) of the index electrocardiogram. The HR is expressed relative to the cohort median score; solid lines indicate the estimated HR and shaded areas the 95% confidence interval, and the horizontal dashed line denotes HR = 1. Gray histograms show the frequency distribution of each score (right *y*-axis). For both scores the association was steeper for 30-day than for 1-year AF; the LVSD score showed a non-monotonic relationship for 1-year AF that peaked near a score of 25 and attenuated at higher scores, where data were sparse and confidence intervals wide. AF, atrial fibrillation; AI-ECG, artificial intelligence-enabled electrocardiography; HR, hazard ratio; LVDD, left ventricular diastolic dysfunction; LVSD, left ventricular systolic dysfunction.

The shape and time dependence of the score–AF association at the continuous level were examined with restricted cubic spline Cox model (Figure 7). For both scores, the association was substantially stronger for AF within 30 days than for AF within 1 year. The LVSD score showed a steep initial rise in hazard, reaching a hazard ratio (HR) of approximately 3.5 for 30-day AF before plateauing; for 1-year AF the association was smaller and non-monotonic, peaking at an HR of about 1.7 near a score of 25 and attenuating at higher scores, where estimates were imprecise and confidence intervals were wide owing to few patients (Figure 7A,B). The LVDD score showed a graded, monotonic dose–response at both time windows, rising to an HR of roughly 2.7 for 30-day AF and plateauing near an HR of 1.3 for 1-year AF (Figure 7C,D).

### 3.9. Sensitivity Analyses

For the sensitivity analysis, we excluded AF events occurring within 7 days (161 events) and within 14 days (194 events) of the index ECG and repeated the 30-day and 1-year analyses in each remaining sample. Both scores remained robustly associated with incident AF at both time windows, with only modest attenuation of discrimination (Supplementary Figure S2A,B). For 1-year AF, the LVSD-score AUROC decreased from 0.713 to 0.694 (7-day) and 0.692 (14-day), and the LVDD-score AUROC from 0.764 to 0.750 and 0.749; for 30-day AF, the corresponding AUROCs were 0.803, 0.763, and 0.769 (LVSD) and 0.817, 0.778, and 0.780 (LVDD). Unadjusted hazard ratios for LVSD and LVDD positivity remained significantly elevated throughout (Supplementary Figure S2C,D): for 1-year AF, the hazard ratio for LVDD positivity was 5.18 (primary), 4.58 (7 days), and 4.62 (14 days), and for LVSD positivity 3.21, 2.53, and 2.48, respectively; estimates for the 30-day window were attenuated but remained significant despite fewer events.

To address potential correlations among multiple ECGs from the same patient, we repeated the 1-year discrimination analysis using a single index ECG per patient (18,984 patients; 581 incident AF, 3.06%). The LVDD score discriminated incident AF with an AUROC of 0.752 (95% CI, 0.732–0.772), not significantly different from the ECG-level estimate (0.764, 0.750–0.778; ΔAUROC 0.011, cluster-bootstrap *p* = 0.124). The LVSD-score AUROC was 0.691 (0.670–0.713), modestly but significantly lower than the ECG-level estimate (0.713, 0.698–0.728; ΔAUROC 0.021, *p* = 0.012), indicating slight overestimation of LVSD discrimination at the ECG level. Both scores remained significant predictors and the LVDD score retained superior discrimination in the patient-level analysis.

### 3.10. Subgroup Analyses

Discrimination for 1-year incident AF was broadly consistent across subgroups, and the LVDD score outperformed the LVSD score in every stratum (Supplementary Figure S3). Discrimination was numerically higher in women than in men (LVSD AUROC 0.754 [95% CI, 0.733–0.775] vs. 0.677 [0.656–0.697]; LVDD 0.819 [0.802–0.836] vs. 0.732 [0.713–0.752]) and in younger than in older patients (age < 60 vs. ≥60 years: LVSD 0.760 [0.734–0.787] vs. 0.664 [0.645–0.682]; and LVDD 0.786 [0.758–0.814] vs. 0.684 [0.664–0.703]). On formal testing of a score × subgroup interaction, effect modification was significant by age for both scores (*p* for interaction < 0.001) and by sex for the LVDD score (*p* < 0.001) but not for the LVSD score (*p* = 0.87), even though the between-subgroup difference in AUROC was statistically significant for both age and sex (all *p* < 0.001); this suggests that the sex difference in LVSD discrimination partly reflects differences in case-mix and score distribution rather than a difference in the per-unit association. Across the comorbidity strata, discrimination was generally similar within each pair (interaction *p* > 0.05) except for congestive heart failure, in which discrimination fell substantially for both scores (LVSD AUROC 0.568 [0.547–0.588]; LVDD 0.631 [0.610–0.652] vs. 0.674 and 0.721 in those without; *p* for interaction < 0.001 for LVDD and 0.04 for LVSD), consistent with near-uniformly elevated scores limiting further stratification. Estimates in the peripheral-artery-disease-positive stratum were imprecise owing to few patients (*n* = 158; 26 events). Full subgroup AUROCs and interaction *p* values are provided in Supplementary Table S5.

To examine whether the reduced discrimination in heart failure reflected loss of risk stratification or compression of the score range, we cross-tabulated 1-year AF incidence across LVSD × LVDD bins separately in patients with and without prevalent heart failure (Supplementary Figure S4). In both strata, AF incidence increased predominantly along the LVDD axis, with a weaker and less consistent gradient across LVSD bins. Within the largest LVSD stratum, AF incidence rose across LVDD bins from 1.2% to 17.6% in patients without heart failure, but from a markedly elevated baseline in those with heart failure (13.7% to 45.2%), compressing the relative range and thereby lowering the AUROC despite a preserved absolute-risk gradient along LVDD.

## 4. Discussion

In a cohort of 18,984 patients (19,593 sinus rhythm index ECGs), two AI-ECG models targeting structural heart disease—LVSD and LVDD—predicted incident AF within one year from a single 12-lead ECG. The two scores had distinct predictive profiles as follows: the LVDD score was the stronger and more durable predictor, with one-year AF incidence of 20.0% in LVDD-positive versus 4.3% in LVDD-negative patients and a one-year AUROC of 0.764, whereas the LVSD score was most informative for near-term events (30-day AUROC 0.803) and attenuated more steeply over follow-up. Combined LVSD/LVDD positivity conferred the highest hazard in the weeks immediately after the index ECG but converged with isolated LVDD positivity by one year. These findings extend prior work in two ways. First, whereas earlier AI-ECG approaches to AF prediction have largely relied on models trained directly to detect an AF-related signature—one of which discriminated incident AF with a one-year ROC-AUC of 0.73 and added value beyond clinical predictors in the UK Biobank [9,10,11,27]—we show that AI-ECG models developed for a different target, left ventricular systolic and diastolic dysfunction, also predict incident AF, with the LVDD model achieving comparable discrimination (one-year AUROC 0.76); this suggests that structural and AF-specific AI-ECG signals capture overlapping but distinct pathways to AF. Second, we show that this predictive performance is markedly time-dependent, being strongest for near-term events and declining over follow-up—a pattern mirrored by the AF-specific model in the UK Biobank, whose discrimination fell from a ROC-AUC of 0.73 at 1 year to 0.69 at 3 years [27]—indicating that AI-ECG AF risk information is dynamic rather than static. Prior studies have developed AF prediction models from the 12-lead ECG using digital biomarkers and deep representation learning [28] or from electronic health records with and without ECG diagnoses [29]. Building on established, regulator-cleared structural AI-ECG scores, our study evaluated their incremental value over a validated clinical score rather than deriving a new multivariable model; prospective, externally validated integration of these scores with clinical variables is a logical next step.

Beyond confirming that structural AI-ECG scores predict incident AF, we found that the LVDD score added discrimination and reclassification beyond the CHA_2_DS_2_-VASc score—a validated clinical benchmark that already encapsulates age, sex, and the major comorbidities associated with AF. This incremental value (an AUROC increase of 0.06 at 30 days and 0.04 at 1 year, with concordant IDI gains) indicates that the diastolic AI-ECG signal captures a pre-clinical atrial substrate not reflected in conventional clinical risk factors. The LVSD score improved overall discrimination but chiefly by down-classifying non-events, with negative event-level reclassification, consistent with its weaker and more short-term association with AF. That combining both scores added nothing beyond CHA_2_DS_2_-VASc plus the LVDD score reinforces that the diastolic score carries the clinically relevant information. These findings support the potential of a single AI-ECG to refine AF risk estimation beyond readily available clinical scores and to help target rhythm surveillance.

The dominance of the LVDD score is biologically coherent as follows: elevated left ventricular filling pressure raises left atrial pressure and promotes atrial stretch, fibrosis, and electrical remodeling—the atrial cardiomyopathy that precedes AF [12,13,14,15,16]—and an AI-ECG score trained to detect elevated E/e′ reflects invasive filling pressures and carries prognostic value [20]. The diastolic signal may therefore index a durable pre-clinical atrial substrate, consistent with its graded and persistent association with AF. The predictive value of both scores was, however, strongly time-dependent, being highest immediately after the index ECG and declining over the year—most steeply for LVSD: excluding events within 7–14 days roughly halved the hazard ratio for LVSD positivity (from 6.58 to 3.6–3.8) while attenuating LVDD proportionally less (from 8.25 to 5.5–6.6), although a substantial short-term association persisted for both. This suggests the LVSD signal partly reflects proximity to clinically manifest or imminent disease rather than long-evolving remodeling, and that both associations persisted after blanking argues against pure reverse causation. Overall, these findings indicate that structural AI-ECG scores behave as dynamic rather than static risk markers, most informative for near-term risk—a pattern echoed by AI-ECG-based heart-failure risk estimation [30].

Because systolic and diastolic dysfunction are mechanistically linked yet distinct, jointly interpreting both AI-ECG models is conceptually attractive, and composite systolic–diastolic AI-ECG models have been used to predict incident heart failure [30] and to track disease during heart-failure therapy [31]. In our data, however, the incremental value of combining the two models for AF prediction was time-limited as follows: dual positivity identified a subgroup at very high short-term risk, but by one year it conferred little additional risk beyond isolated LVDD positivity, as shown concordantly by the time-varying Cox, Aalen additive hazards, and restricted mean survival time analyses. For the specific task of one-year AF prediction, the LVDD model therefore carries most of the information, whereas the combined model is most useful for flagging patients at elevated imminent risk.

These findings suggest that a single, inexpensive sinus-rhythm ECG could help identify patients in whom intensified AF surveillance—such as targeted ambulatory monitoring—is most likely to be productive, particularly shortly after acquisition and in patients with combined systolic–diastolic dysfunction; the short-term concentration of risk aligns well with the days-to-weeks window of Holter or patch monitoring [10]. Positioning these scores as time-aware markers could complement clinical AF risk scores [7,8]. These implications are, however, hypothesis-generating: as a retrospective, single-center study without systematic rhythm monitoring or external validation, our results support further investigation of AI-ECG-guided AF surveillance but do not justify clinical implementation. Prospective, externally validated studies testing whether AI-ECG-triggered monitoring increases AF detection and improves outcomes are needed before routine use.

Discrimination of both AI-ECG scores was lower in patients with prevalent heart failure (LVSD AUROC 0.57, LVDD 0.63) than in those without (0.67 and 0.72). This is an expected consequence of how the scores are generated: because the models are trained to detect systolic and diastolic dysfunction, patients with established heart failure carry near-uniformly high scores, and this compression of the score range—together with a high baseline burden of AF risk factors—lowers the AUROC even though the scores remain valid markers of structural disease. Reduced discrimination does not, however, negate clinical value. Within the heart-failure subgroup, higher LVDD scores still identified higher absolute AF risk (13.7% to 45.2% across LVDD bins in the largest LVSD stratum; Supplementary Figure S4), and in the overall cohort the LVDD score improved discrimination and reclassification beyond the CHA_2_DS_2_-VASc score, which already incorporates heart failure—indicating that the signal is not merely a restatement of a heart-failure diagnosis. The scores are therefore most useful for AF risk stratification in patients without recognized heart failure, in whom occult structural dysfunction is otherwise unmeasured, while offering limited incremental discrimination in those already known to be at high risk.

Several limitations should be considered. First, this was a single-center, retrospective study, which may limit generalizability. Second, and most important, incident AF was ascertained from clinically obtained 12-lead ECGs and corresponding EHR diagnoses rather than from systematic or ambulatory rhythm monitoring. Consequently, paroxysmal or asymptomatic AF occurring outside the healthcare setting may have gone undetected, and outcome detection depended on the frequency of clinical encounters; because patients with abnormal AI-ECG scores likely undergo more frequent follow-up, AF may have been preferentially detected among score-positive patients—contributing to the strong short-term associations—while being under-ascertained in score-negative patients with fewer encounters. Consistent with this, patients in the AF group underwent more follow-up ECGs than controls (mean 7.0 vs. 2.95 per patient), although the number of ECGs could not be disaggregated by AI-ECG risk group. A related consequence is that we cannot fully distinguish genuine prediction of future AF from identification of ECG signatures of already present but undiagnosed paroxysmal AF, as the scores may partly capture atrial remodeling coexisting with occult AF. Our requirement for concordance between the ECG rhythm and the clinical diagnosis, the attenuation but persistence of the associations after 7- and 14-day blanking, and the graded dose–response argue against a wholly artifactual effect; nevertheless, early events may still include some subclinical AF. This detection bias is inherent to a retrospective, ECG-based design—although it has limited bearing on clinical utility, since both scenarios point to closer rhythm surveillance—and could be fully resolved only by a prospective study with systematic baseline and ambulatory monitoring. Third, adjustment was limited and residual confounding cannot be excluded, and death was not modeled as a competing risk. Finally, the prespecified cutoffs were optimized for detecting structural dysfunction rather than for AF prediction, and their transportability to other populations is uncertain; estimates in the highest score bins were imprecise owing to sparse data.

## 5. Conclusions

Structural AI-ECG LVSD and LVDD scores from a single sinus-rhythm ECG predict incident AF in a time-dependent manner, with the strongest performance shortly after acquisition and more durable discrimination for LVDD. A single AI-ECG may help target short-term AF surveillance, particularly in patients with combined systolic–diastolic dysfunction. These findings should be confirmed in prospective, externally validated studies before clinical adoption.

## Figures and Tables

**Table 1:** Baseline characteristics by incident AF at one year.

Variable	All Subjects	No AF	Incident AF	*p* Value
*N*	19,593	18,403	1190	
Age, years, median (IQR)	58 (48–68)	58 (47–67)	67 (58–76)	<0.001
Male sex, *n* (%)	9706 (49.5)	9041 (49.1)	665 (55.9)	<0.001
Underlying disease, *n* (%)				
Hypertension	8073 (41.2)	7290 (39.6)	783 (65.8)	<0.001
Diabetes	2746 (14.0)	2450 (13.3)	296 (24.9)	<0.001
Coronary artery disease	5312 (27.1)	4904 (26.6)	408 (34.3)	<0.001
Cerebrovascular disease	1277 (6.5)	1112 (6.0)	165 (13.9)	<0.001
Congestive heart failure	3677 (18.8)	2847 (15.5)	830 (69.7)	<0.001
Chronic kidney disease	1001 (5.1)	810 (4.4)	191 (16.1)	<0.001
Peripheral artery disease	158 (0.8)	132 (0.7)	26 (2.2)	<0.001
Clinical risk score, median (IQR)				
CHA_2_DS_2_-VASc score	1.0 (1.0–3.0)	1.0 (1.0–3.0)	3.0 (2.0–4.0)	<0.001
ECG features, median (IQR)				
Heart rate, bpm	71 (63–81)	71 (63–81)	68 (60–79)	<0.001
PR interval, ms	164 (149–181)	164 (149–180)	175 (156–199)	<0.001
QRS duration, ms	94 (87–101)	93 (87–100)	98 (91–108)	<0.001
QTc interval, ms	431 (414–450)	431 (413–449)	444 (424–469)	<0.001
T-wave axis, °	40 (23–57)	40 (23–56)	45 (22–64)	<0.001
AI–ECG results				
LVSD score, median (IQR)	0.2 (0.1–0.7)	0.2 (0.1–0.6)	0.9 (0.3–3.6)	<0.001
LVDD score, median (IQR)	3.1 (1.1–9.2)	2.9 (1.0–8.1)	14.3 (5.1–33.0)	<0.001
LVSD ≥ 9.7, *n* (%)	1206 (6.2)	1012 (5.5)	194 (16.3)	<0.001
LVDD ≥ 20.8, *n* (%)	2248 (11.5)	1798 (9.8)	450 (37.8)	<0.001
LVSD < 9.7 and LVDD < 20.8, *n* (%)	16,888 (86.2)	16,193 (88.0)	695 (58.4)	<0.001
LVSD ≥ 9.7 and LVDD ≥ 20.8, *n* (%)	749 (3.8)	600 (3.3)	149 (12.5)	<0.001

Values are presented per index ECG; the incident AF group comprised 581 unique patients contributing 1190 index ECGs, whereas each control contributed a single ECG. AF, atrial fibrillation; IQR, interquartile range; ECG, electrocardiography; QTc, corrected QT; AI–ECG, artificial intelligence-enabled electrocardiography; LVSD, left ventricular systolic dysfunction; LVDD, left ventricular diastolic dysfunction.

**Table 2:** AI–ECG discrimination for incident AF across follow-up durations.

	AF Event	LVSD	LVDD	
Follow-Up Durations	*n* (%)	AUROC with 95% CI	AUROC with 95% CI	*p* Value
30 days	265 (1.4)	0.803 (0.777–0.829)	0.817 (0.791–0.843)	0.289
90 days	480 (2.4)	0.771 (0.750–0.791)	0.794 (0.774–0.815)	0.026
180 days	754 (3.8)	0.731 (0.712–0.749)	0.771 (0.754–0.788)	<0.001
270 days	985 (5.0)	0.725 (0.709–0.740)	0.765 (0.750–0.780)	<0.001
365 days	1190 (6.1)	0.713 (0.698–0.728)	0.764 (0.750–0.778)	<0.001

AI–ECG, artificial intelligence-enabled electrocardiography; AF, atrial fibrillation; LVSD, left ventricular systolic dysfunction; AUROC, area under the receiver operating characteristics curve; LVDD, left ventricular diastolic dysfunction.

## Data Availability

The patient-level data supporting the findings of this study cannot be shared publicly because of institutional review board policy and patient privacy protections. The AI-ECG models and inference outputs used in this study may be made available from the corresponding author upon reasonable request.

## Supplementary Materials

The following supporting information can be downloaded at https://www.mdpi.com/article/10.3390/diagnostics16182887/s1, Supplementary Table S1. Baseline characteristics by incident AF at 30-day; Supplementary Table S2. Discrimination of the CHA2DS2-VASc score alone and with AI-ECG scores added, for incident AF at 30 days and 1 year; Supplementary Table S3. Reclassification (category-free NRI and IDI) of AI-ECG scores added to the CHA2DS2-VASc score, for incident AF at 30 days and 1 year; Supplementary Table S4. Hazard ratios of joint AI–ECG LVSD × AI–ECG LVDD group (both negative; LVSD-positive only; LVDD-positive only; both positive) for incident AF across follow-up durations; Supplementary Table S5. Subgroup discrimination of the AI-ECG LVSD and LVDD scores for one-year incident AF, with tests of between-subgroup difference and interaction; Supplementary Figure S1. Instantaneous hazard ratios for the joint AI-ECG LVSD × LVDD groups across follow-up, estimated from a single time-varying Cox model with a group × log(time) interaction; Supplementary Figure S2. Blanking-period sensitivity analysis for the AI-ECG LVSD and LVDD scores and incident AF; Supplementary Figure S3. Subgroup analysis of the discrimination (AUROC) of the AI-ECG LVSD and LVDD scores for 1-year incident AF; Supplementary Figure S4. One-year AF incidence across joint LVSD × LVDD score bins, stratified by prevalent congestive heart failure.

## Abbreviations

The following abbreviations are used in this manuscript:

AF	Atrial fibrillation
AI-ECG	Artificial intelligence-enabled electrocardiography
AUC	Area under the curve
AUROC	Area under the receiver operating characteristic curve
CHA2DS2-VASc	Congestive heart failure, hypertension, age, diabetes, stroke, vascular disease, sex category (clinical risk score)
CI	Confidence interval
ECG	Electrocardiography
E/e’	Ratio of early mitral inflow velocity to early diastolic mitral annular velocity
HR	Hazard ratio
ICD-10	International Classification of Diseases, 10th Revision
IQR	Interquartile range
IRB	Institutional review board
LVDD	Left ventricular diastolic dysfunction
LVSD	Left ventricular systolic dysfunction
QTc	Corrected QT interval
RMST	Restricted mean survival time
ROC	Receiver operating characteristic
