# Supplementary material for: Artificial Intelligence-Enabled Electrocardiography Model for Left Ventricular Systolic and Diastolic Dysfunction Predict Incident Atrial Fibrillation in Patients with Sinus Rhythm: A Time-Dependent Analysis
Source: Diagnostics (Basel). 2026 Sep 8;16(18):2887. doi: 10.3390/diagnostics16182887 (PMC13605585; doi:10.3390/diagnostics16182887)
Supplement: Supplementary file 1 [file diagnostics-16-02887-s001.zip › diagnostics-4527443-supplementary.pdf]

Supplementary Table S1. Baseline characteristics by incident AF at 30-day

Variable	All subjects	No AF	Incident AF	*P* value
N	19,593	19,328	265	
Age, years, median (IQR)	58 (48-68)	58 (48-68)	72 (60-82)	<0.001
Male sex, n (%)	9706 (49.5)	9576 (49.5)	130 (49.1)	0.924
Underlying disease, n (%)				
Hypertension	8073 (41.2)	7915 (41.0)	158 (59.6)	<0.001
Diabetes	2746 (14.0)	2684 (13.9)	62 (23.4)	<0.001
Coronary artery disease	5312 (27.1)	5209 (27.0)	103 (38.9)	<0.001
Cerebrovascular disease	1277 (6.5)	1223 (6.3)	54 (20.4)	<0.001
Congestive heart failure	3677 (18.8)	3495 (18.1)	182 (68.7)	<0.001
Chronic kidney disease	1001 (5.1)	946 (4.9)	55 (20.8)	<0.001
Peripheral artery disease	158 (0.8)	150 (0.8)	8 (3.0)	<0.001
Clinical risk score, median (IQR)				
CHA_2DS_2-VASc score	1.0 (1.0-3.0)	1.0 (1.0-3.0)	3.0 (2.0-5.0)	<0.001
ECG features, median (IQR)				
Heart rate, bpm	71 (63-81)	71 (63-81)	79 (65-97)	<0.001
PR interval, ms	164 (149-181)	164 (149-181)	170 (148-193)	0.021
QRS duration, ms	94 (87-101)	94 (87-101)	98 (89-107)	<0.001
QTc interval, ms	431 (414-450)	431 (414-449)	459 (436-493)	<0.001
T-wave axis, °	40 (23-57)	40 (23-57)	47 (10-67.2)	0.083
AI–ECG results				
LVSD score, median (IQR)	0.2 (0.1-0.7)	0.2 (0.1-0.7)	2.6 (0.6-13.8)	<0.001
LVDD score, median (IQR)	3.1 (1.1-9.2)	3.1 (1.1-8.8)	21.5 (9.7-45.8)	<0.001
LVSD ≥9.7, n (%)	1206 (6.2)	1128 (5.8)	78 (29.4)	<0.001
LVDD ≥20.8, n (%)	2248 (11.5)	2113 (10.9)	135 (50.9)	<0.001
LVSD <9.7 and LVDD <20.8, n (%)	16888 (86.2)	16775 (86.8)	113 (42.6)	<0.001
LVSD ≥9.7 and LVDD ≥20.8, n (%)	749 (3.8)	688 (3.6)	61 (23.0)	<0.001

Values are presented per index ECG; the incident AF group comprised 581 unique patients contributing 1,190 index ECGs, whereas each control contributed a single ECG.

AF, atrial fibrillation; IQR, interquartile range; ECG, electrocardiography; QTc, corrected QT; AI–ECG, artificial intelligence–enabled electrocardiography; LVSD, left ventricular systolic dysfunction; LVDD, left ventricular diastolic dysfunction.

Supplementary Table S2. Discrimination of the CHA_2DS_2-VASc score alone and with AI-ECG scores added, for incident AF at 30 days and 1 year.

Outcome	Model	AUROC (95% CI)	ΔAUROC	*P* value
30-day AF	CHA2DS2-VASc	0.774 (0.746–0.802)	ref	—
	+ LVSD	0.822 (0.797–0.847)	+0.048	<0.001
	+ LVDD	0.834 (0.810–0.858)	+0.060	<0.001
	+ LVSD + LVDD	0.835 (0.811–0.859)	+0.062	<0.001
1-year AF	CHA2DS2-VASc	0.746 (0.732–0.760)	ref	—
	+ LVSD	0.774 (0.761–0.787)	+0.028	<0.001
	+ LVDD	0.783 (0.770–0.796)	+0.037	<0.001
	+ LVSD + LVDD	0.783 (0.770–0.796)	+0.037	<0.001

ΔAUROC and DeLong P are versus CHA2DS2-VASc alone. AUROC, area under the receiver operating characteristic curve; LVSD/LVDD, AI-ECG left ventricular systolic/diastolic dysfunction score.

Supplementary Table S3. Reclassification (category-free NRI and IDI) of AI-ECG scores added to the CHA_2DS_2-VASc score, for incident AF at 30 days and 1 year.

Outcome	Added to CHA2DS2-VASc	NRI, total (95% CI)	NRI, events	NRI, non-events	IDI (95% CI)
30-day AF	+ LVSD	0.485 (0.377–0.599)	−0.389	0.873	0.015 (0.009–0.021)
	+ LVDD	0.744 (0.627–0.865)	0.049	0.695	0.024 (0.017–0.031)
	+ LVSD + LVDD	0.811 (0.691–0.935)	0.087	0.725	0.026 (0.018–0.034)
1-year AF	+ LVSD	0.254 (0.208–0.301)	−0.595	0.849	0.007 (0.004–0.009)
	+ LVDD	0.550 (0.492–0.607)	−0.081	0.631	0.032 (0.027–0.037)
	+ LVSD + LVDD	0.542 (0.479–0.599)	−0.077	0.619	0.032 (0.027–0.037)

Category-free NRI and IDI with 95% CIs from 1,000 bootstrap resamples. Positive values indicate improved reclassification. IDI, integrated discrimination improvement; LVSD/LVDD, AI-ECG left ventricular systolic/diastolic dysfunction score; NRI, net reclassification improvement.

Supplementary Table S4. Hazard ratios of joint AI–ECG LVSD × AI–ECG LVDD group (both negative; LVSD-positive only; LVDD-positive only; both positive) for incident AF across follow-up durations

	Follow-up durations				
Total N = 19,593	30-day	90-day	180-day	270-day	365-day
Total AF event, n (%)	265 (1.4)	480 (2.4)	754 (3.8)	985 (5.0)	1,190 (6.1)
Both negative (n=16,888)					
AF event, n (%)	113 (0.7)	235 (1.4)	416 (2.5)	560 (3.3)	695 (4.1)
HR with 95% CI	Reference				
LVSD positive only (n=457)					
AF event, n (%)	17 (3.7)	25 (5.5)	34 (7.4)	40 (8.8)	45 (9.8)
HR with 95% CI	5.67 (3.41-9.44)	4.04 (2.67-6.10)	3.12 (2.20-4.43)	2.74 (1.99-3.78)	2.50 (1.85-3.38)
LVDD positive only (n=1,499)					
AF event, n (%)	74 (4.9)	134 (8.9)	194 (12.9)	254 (16.9)	301 (20.1)
HR with 95% CI	7.53 (5.62-10.10)	6.68 (5.40-8.25)	5.57 (4.70-6.61)	5.51 (4.75-6.40)	5.35 (4.67-6.12)
Both positive (n=749)					
AF event, n (%)	61 (8.1)	86 (11.5)	110 (14.7)	131 (17.5)	149 (19.9)
HR with 95% CI	12.75 (9.34-17.41)	8.85 (6.91-11.33)	6.50 (5.27-8.03)	5.84 (4.83-7.06)	5.42 (4.54-6.46)

AI–ECG, artificial intelligence–enabled electrocardiography; LVSD, left ventricular systolic dysfunction; AF, atrial fibrillation; HR, hazard ratio.

Supplementary Table S5. Subgroup discrimination of the AI-ECG LVSD and LVDD scores for one-year incident AF, with tests of between-subgroup difference and interaction.

Subgroup	Score	AUROC (present)	AUROC (absent)	AUROC diff	P value	aOR P value
Female sex	LVSD	0.754	0.676	0.078	<0.001	0.866
Female sex	LVDD	0.819	0.732	0.087	<0.001	<0.001
Age <60	LVSD	0.760	0.664	0.097	<0.001	<0.001
Age <60	LVDD	0.786	0.684	0.102	<0.001	<0.001
Hypertension	LVSD	0.679	0.747	-0.068	<0.001	0.006
Hypertension	LVDD	0.738	0.747	-0.009	0.562	0.713
Diabetes	LVSD	0.687	0.708	-0.020	0.254	0.912
Diabetes	LVDD	0.742	0.757	-0.014	0.388	0.302
Coronary artery disease	LVSD	0.709	0.709	0.000	0.985	0.288
Coronary artery disease	LVDD	0.765	0.760	0.005	0.747	0.355
Cerebrovascular disease	LVSD	0.695	0.710	-0.015	0.483	0.130
Cerebrovascular disease	LVDD	0.736	0.761	-0.025	0.283	0.106
Congestive heart failure	LVSD	0.568	0.674	-0.106	<0.001	0.039
Congestive heart failure	LVDD	0.631	0.721	-0.089	<0.001	<0.001
Chronic kidney disease	LVSD	0.689	0.697	-0.008	0.711	0.577
Chronic kidney disease	LVDD	0.722	0.751	-0.029	0.188	0.144
Peripheral artery disease	LVSD	0.662	0.713	-0.051	0.404	0.017
Peripheral artery disease	LVDD	0.736	0.763	-0.027	0.563	0.892

Values are the area under the receiver operating characteristic curve (AUROC) for one-year incident AF, computed separately within each subgroup. For each characteristic, "AUROC (present)" is the AUROC in patients with the characteristic present (female sex; age < 60 years; comorbidity present), and "AUROC (absent)" is the AUROC in the complementary group (male sex; age ≥ 60 years; comorbidity absent); "AUROC diff" is their difference (present − absent). The "P value" tests the between-subgroup difference in AUROC. The "aOR P value" is the P value for the interaction between the continuous score and the subgroup in a logistic regression model for incident AF (adjusted for age and sex where applicable), testing whether the per-unit association of the score with AF differs by subgroup (effect modification). A significant AUROC difference reflects a difference in discrimination, which may arise from differences in case-mix, event rate, or score distribution and does not necessarily indicate a difference in the underlying score–AF association; the interaction test addresses the latter.

AUROC, area under the receiver operating characteristic curve; aOR, adjusted odds ratio; LVSD/LVDD, AI-ECG left ventricular systolic/diastolic dysfunction score.

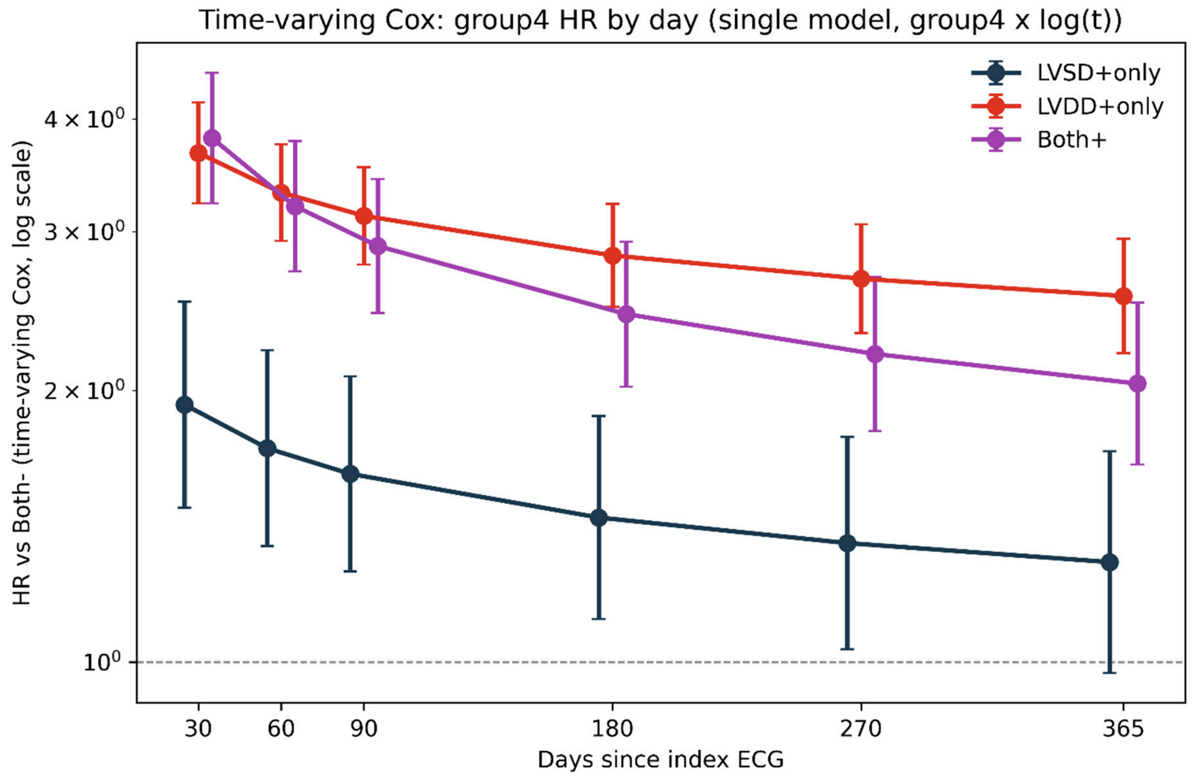

Supplementary Figure S1. *Instantaneous hazard ratios for the joint AI-ECG LVSD × LVDD groups across follow-up, estimated from a single time-varying Cox model with a group × log(time) interaction.*

Hazard ratios for the LVSD-positive-only, LVDD-positive-only, and both-positive groups relative to the both-negative reference are shown at 30, 60, 90, 180, 270, and 365 days since the index electrocardiogram; symbols denote point estimates and error bars the 95% confidence intervals on a log scale, and the dashed line indicates a hazard ratio of 1. Unlike the windowed cumulative hazard ratios in Figure 5A, this model estimates the instantaneous hazard ratio and assumes a log-linear change over time. The instantaneous hazard ratio for the both-positive group exceeded that of the LVDD-positive-only group only in the first weeks and fell to or below it thereafter, whereas the LVSD-positive-only group remained the lowest throughout.

AF, atrial fibrillation; AI-ECG, artificial intelligence–enabled electrocardiography; HR, hazard ratio; LVSD, left ventricular systolic dysfunction; LVDD, left ventricular diastolic dysfunction.

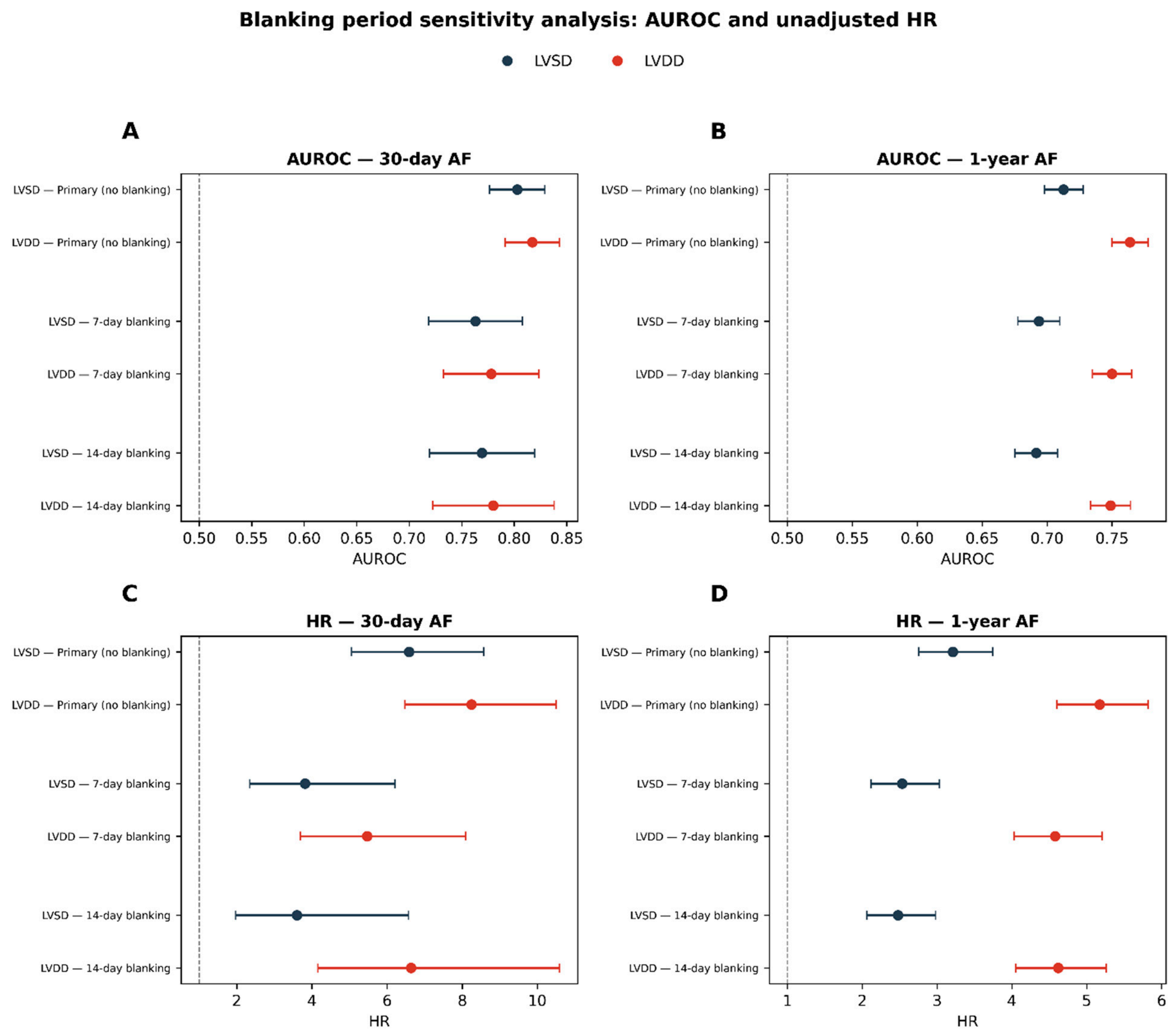

Supplementary Figure S2. *Blanking-period sensitivity analysis for the AI-ECG LVSD and LVDD scores and incident AF.*

*Discrimination (AUROC) of the continuous AI-ECG LVSD (navy) and LVDD (red) scores for AF occurring within 30 days (**A**) and within 1 year (365 days) (**B**), and unadjusted hazard ratios (HR) for LVSD and LVDD positivity at the prespecified cutoffs (LVSD ≥ 9.7; LVDD ≥ 20.8) for AF within 30 days (**C**) and within 1 year (**D**). Each panel shows the primary analysis (no blanking) and the analyses repeated after excluding AF events occurring within 7 days or within 14 days of the index electrocardiogram. Symbols denote point estimates and horizontal bars the 95% confidence intervals; the vertical dashed line indicates the value expected by chance (AUROC = 0.50 in A and B; HR = 1.0 in C and D). Both scores remained associated with incident AF at both time windows after blanking, with only modest attenuation, indicating that the associations were not merely due to detection of imminent AF.*

AF, atrial fibrillation; AI-ECG, artificial intelligence–enabled electrocardiography; AUROC, area under the receiver operating characteristic curve; HR, hazard ratio; LVDD, left ventricular diastolic dysfunction; LVSD, left ventricular systolic dysfunction.

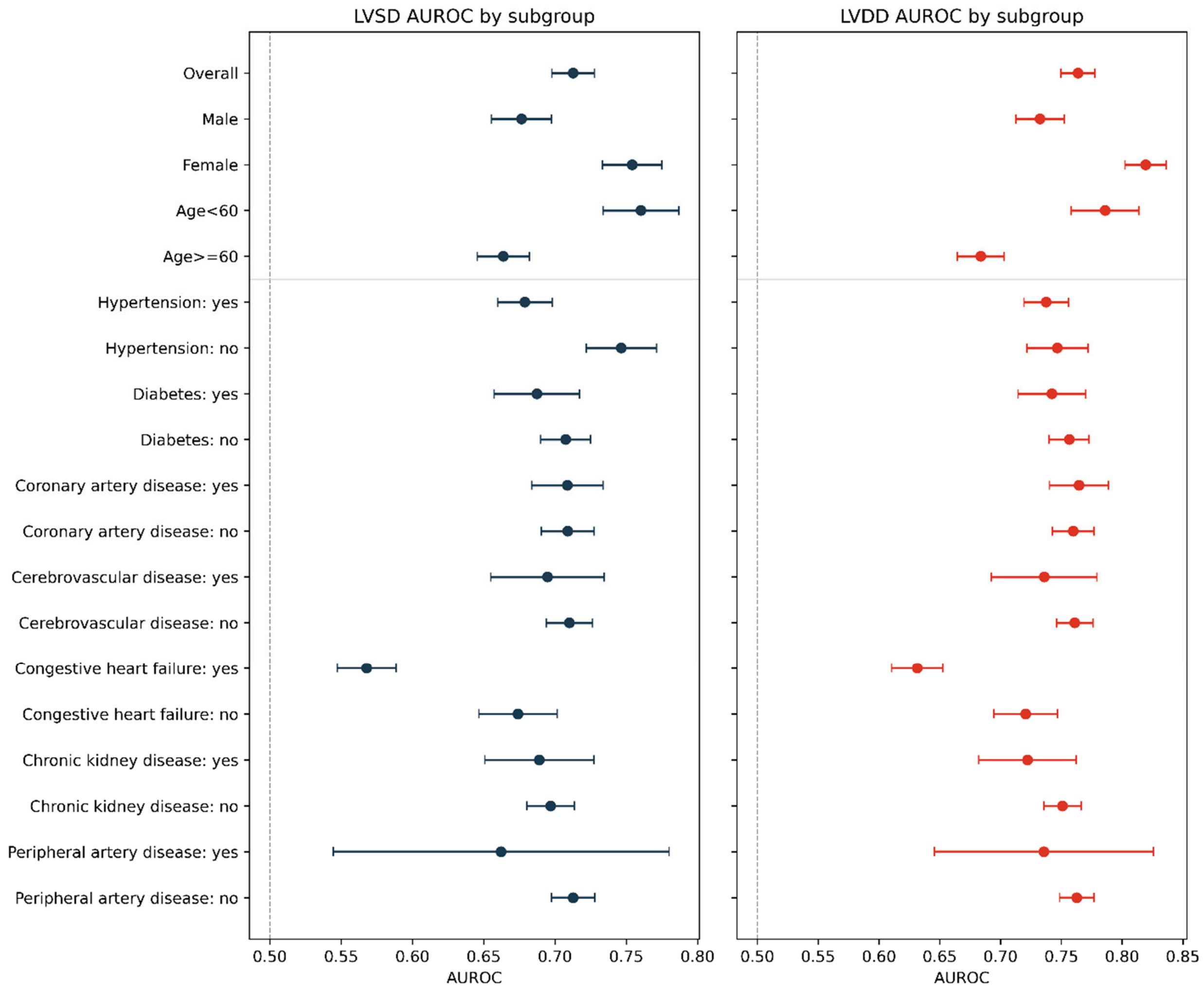

Supplementary figure S3. *Subgroup analysis of the discrimination (AUROC) of the AI-ECG LVSD and LVDD scores for 1-year incident AF.*

AUROC of the AI-ECG LVSD score (left) and LVDD score (right) for incident atrial fibrillation within 1 year of the index electrocardiogram, shown overall and within subgroups defined by sex, age (< 60 vs ≥ 60 years), and prevalent comorbidities (hypertension, diabetes, coronary artery disease, cerebrovascular disease, congestive heart failure, chronic kidney disease, and peripheral artery disease). Symbols denote point estimates and horizontal bars the 95% confidence intervals; the vertical dashed line indicates a discrimination of 0.50 (no better than chance). The LVDD score showed higher discrimination than the LVSD score in nearly all subgroups, and discrimination was consistently lower in patients with prevalent congestive heart failure.

AF, atrial fibrillation; AI-ECG, artificial intelligence–enabled electrocardiography; AUROC, area under the receiver operating characteristic curve; LVDD, left ventricular diastolic dysfunction; LVSD, left ventricular systolic dysfunction.

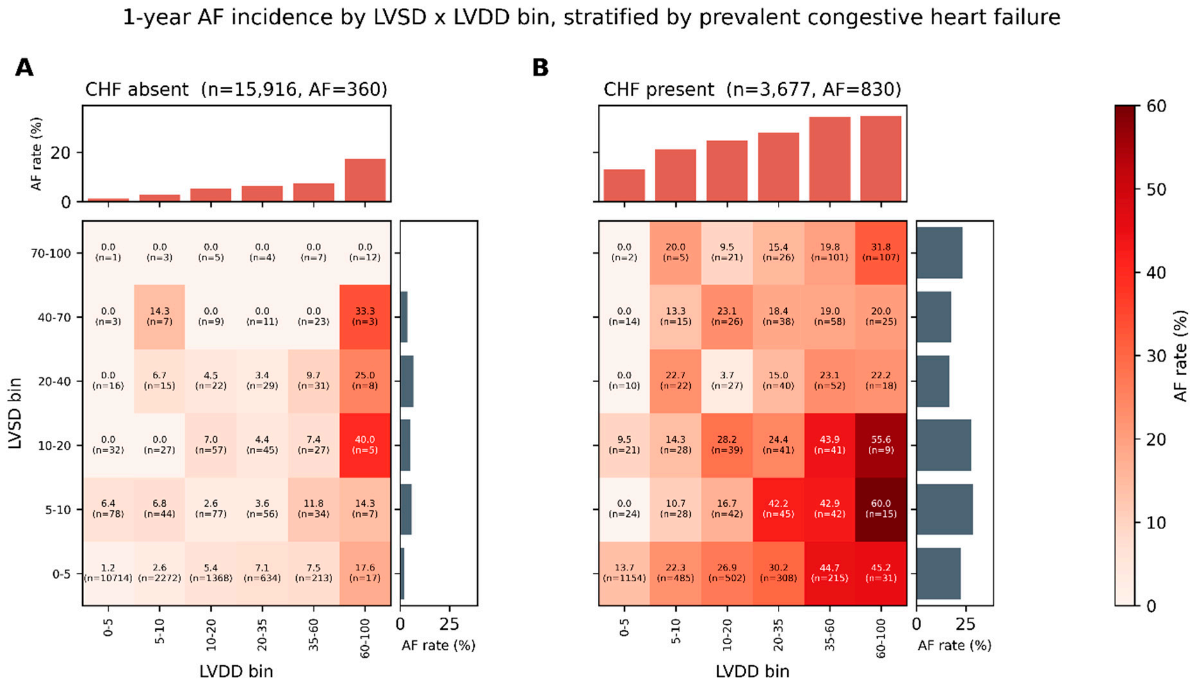

Supplementary Figure S4. *One-year AF incidence across joint LVSD × LVDD score bins, stratified by prevalent congestive heart failure. Heatmaps show observed 1-year AF incidence (%) within each LVSD (rows) × LVDD (columns) bin in patients without (A) and with (B) prevalent heart failure; cell labels give the incidence and number of patients, and marginal bars show incidence by LVDD (top) and LVSD (right) bin. In both strata AF incidence increased predominantly along the LVDD axis, with a weaker gradient across LVSD bins, and from a substantially higher baseline in patients with heart failure. AF, atrial fibrillation; LVSD/LVDD, AI-ECG left ventricular systolic/diastolic dysfunction score.*
